# E-Mental Health in Older Age

**DOI:** 10.1192/j.eurpsy.2022.113

**Published:** 2022-09-01

**Authors:** U. Hegerl

**Affiliations:** Goethe University, Psychiatry, Psychosomatics, And Psychotherapy, Frankfurt, Germany

**Keywords:** e-mental health, depression, self-management, CBT

## Abstract

E-Mental Health in older age Ulrich Hegerl, Caroline Oehler Department of Psychiatry, Psychosomatics, and Psychotherapy, Goethe Universität Frankfurt/M, Germany European Alliance against Depression e.V. (www.EAAD.net) The implementation and uptake of digital tools for self management or psychotherapy for people suffering from depression or other mental disorders has gained momentum during the Covid-19 pandemia. While studies using waiting list or treatment as usual control groups are of limited value, meta-analyses of RCTs with face-to-face psychotherapy as control condition have found a comparable antidepressant effect, especially when the interventions were provided together with professional guidance. The iFightDepression-tool offered by the European Alliance against Depression (EAAD) is available in 10 different languages and is broadly used in several European countries. Data will be presented concerning the attitude of older people concerning iCBT and also concerning effects of age, guidance, and gender on both adherence to the iFightDepression-tool and antidepressant effects.

**Disclosure:**

No significant relationships.

